# Advanced liver disease in Russian children and adolescents with chronic hepatitis C

**DOI:** 10.1111/jvh.13093

**Published:** 2019-04-07

**Authors:** Anna Turkova, Galina V. Volynets, Siobhan Crichton, Tamara A. Skvortsova, Victoria N. Panfilova, Natalia V. Rogozina, Anatoly I. Khavkin, Elena L. Tumanova, Giuseppe Indolfi, Claire Thorne

**Affiliations:** ^1^ MRC Clinical Trials Unit at UCL Institute of Clinical Trials and Methodology University College London London UK; ^2^ Paediatric Infectious Diseases Department Great Ormond Street Hospital London UK; ^3^ Veltischev Research and Clinical Institute of Pediatrics Pirogov Russian National Research Medical University Moscow Russia; ^4^ Federal State Autonomous Institution National Scientific and Practical Center of Children's Health of the Ministry of Health of the Russian Federation Moscow Russia; ^5^ Centre of Children's Gastroenterology Morozovskaya Children's City Clinical Hospital Moscow Russia; ^6^ Department of Pediatrics Institute of Postgraduate Education Krasnoyarsk State Medical University of the Ministry of Health of the Russian Federation Krasnoyarsk Russia; ^7^ Department of Congenital Infections Pediatric Research and Clinical Center for Infectious Diseases Saint‐Petersburg Russia; ^8^ Department of Pathological Anatomy of Pediatric Faculty Pirogov Russian National Research Medical University Moscow Russia; ^9^ Pediatric and Liver Unit Meyer Children's University Hospital of Florence Florence Italy; ^10^ University College London Institute of Child Health University College London London UK

**Keywords:** adolescent, biopsy, child, chronic hepatitis C, elastography, liver fibrosis, Russia

## Abstract

Russia has one of the highest prevalences of paediatric chronic hepatitis C infection (CHC). Our aim was to provide a detailed characterization of children and adolescents with CHC including treatment outcomes. Thus, an observational study of children with CHC aged <18 years was conducted in three hepatology centres from November 2014 to May 2017. Of 301 children (52% male), 196 (65%) acquired HCV vertically, 70 (23%) had a history of blood transfusion or invasive procedures, 1 injecting drug use and 34 (11%) had no known risk factors. Median age at HCV diagnosis was 3.1 [interquartile range, IQR 1.1, 8.2] and 10.8 [7.4, 14.7] at last follow‐up. The most common genotype was 1b (51%), followed by 3 (37%). Over a quarter of patients (84, 28%) had raised liver transaminases. Of 92 with liver biopsy, 38 (41%) had bridging fibrosis (median age 10.4 [7.1, 14.1]). Of 223 evaluated by transient elastography, 67 (30%) had liver stiffness ≥5.0 kPa. For each year, increase in age mean stiffness increased by 0.09 kPa (95% CI 0.05, 0.13, *P* < 0.001). There was significant correlation between liver stiffness and biopsy results (Tau‐b = 0.29, *P* = 0.042). Of 205 treated with IFN‐based regimens, 100 (49%) had SVR24. Most children (191, 93%) experienced adverse reactions, leading to treatment discontinuation in 6 (3%). In conclusion, a third of children acquired HCV via nonvertical routes and a substantial proportion of those with liver biopsy had advanced liver disease. Only half of children achieved SVR24 with IFN‐based regimens highlighting the need for more effective and better‐tolerated treatments with direct‐acting antivirals. Further studies are warranted in Russia on causes and prevention of nonvertical transmission of HCV in children.

**Abbreviations**
ALT: alanine aminotransferase
AST: aspartate aminotransferase
CHC: chronic hepatitis C
GT: genotype
HAZ: height‐for‐age *z*‐scores
IQR: interquartile range
RBV: ribavirin
SD: standard deviation
WHO: World Health Organization

## 1 INTRODUCTION

Chronic hepatitis C (CHC) remains one of the major causes of cirrhosis, hepatocellular carcinoma and end‐stage liver disease worldwide. The World Health Assembly identified hepatitis C as a global health priority and in 2016 endorsed the Global Health Sector Strategy with ambitious targets to reduce new infections by 90% and deaths by 65% by 2030.^1^, ^2^

The World Health Organization (WHO) estimates that there are 71 million people living with hepatitis C virus (HCV) infection (based on HCV viraemia data), accounting for 1% of the global population; only 20% of those infected know about their diagnosis. Of those who are chronically infected, 15%‐30% progress to cirrhosis in 20 years and 399 000 people die annually.^3^, ^4^ The WHO European region, which includes the whole of Europe and Central Asia, is one of the most affected global regions with an estimated chronic HCV infection prevalence of 1.5% and incidence of 62 per 100 000 population per year.^4^ Reliable paediatric estimates are lacking, as HCV infection in children remains largely undiagnosed.

Russia has one of the highest burdens of HCV infection globally with an estimated seroprevalence of 4.3% and the highest absolute number of chronic infections of 3.2 million.^5^ In 2013, a CHC prevalence of 336 per 100 000 population was reported in the HCV national surveillance system, reaching 670 per 100 000 in some regions; overall CHC incidence was 40 per 100 000 per year but varied widely across regions, from 13 to 68.^6^ Over the last few years, the reported CHC incidence has gradually decreased to 35‐36 per 100 000 in 2016‐2017.^7^ Genotype (GT) 1b is the most prevalent genotype accounting for 55% of all cases, followed by GT3 (35%).^8^

In 2013, the reported CHC prevalence was 14 per 100 000 children aged 1‐14,^6^ with the corresponding incidence of 1.75 per 100 000 children.^9^ Similar to adults, there is a downward trend observed over the last years with the reported incidence of 1.33‐1.44 per 100 000 children in 2016‐2017.^7^ Vertical transmission was the most common mode of acquisition accounting for two‐thirds of new infections, with anti‐HCV prevalence among pregnant women reported as 1.3% in 2011‐2012.^6^ Route of transmission was not determined for 21% of all infected children aged ≤14 years.^6^

Our aim was to provide a detailed characterization of children and adolescents with chronic HCV with regard to mode of acquisition, HCV genotype, clinical status, type of treatment and treatment outcomes in three paediatric hepatology centres in Russia in the pre direct‐acting antivirals (DAA) era.

## 2 MATERIALS AND METHODS

An observational study of children and adolescents with chronic HCV followed up in three Russian hepatology centres was conducted in November 2014 to July 2015. The participating centres covered three distinct geographical regions: the Children's Scientific and Clinical Center of Infectious Diseases, St. Petersburg, the National Scientific and Practical Center of Children's Health, Moscow, and the Krasnoyarsk State Medical University.

A total of 100 children seen consecutively in each centre and meeting eligibility criteria were included.^10^ The eligibility criteria were diagnosis with chronic HCV aged <18 years and being in routine follow‐up at the participating centres.

Pseudonymized individual‐patient data were retrospectively collected according to a standard protocol using the secure, web‐based REDCap electronic data capture tool.^11^ Additional data collection on treatment response and adverse events for the sub‐group of children still undergoing treatment in July 2015 was conducted prospectively until May 2017, to obtain treatment outcome data.

Ethics approval for the study was obtained from the National Scientific and Practical Center of Children's Health of the Ministry of Health of the Russian Federation, Moscow.

### 2.1 Definitions

Chronic HCV was defined as detectable HCV RNA in two or more blood samples at least 6 months apart. For children not vertically infected, duration of diagnosed infection was calculated as time from date of diagnosis.

The mode of transmission was reported by participating clinics as vertical (mother is known to have HCV), suspected healthcare‐associated infection (history of receipt of the blood products or invasive procedures in healthcare settings and no maternal history of HCV), injecting drug use or unknown.

Normal ranges of alanine aminotransferase (ALT) and aspartate aminotransferase (AST) are age‐dependent and changed during the study period. We used the cut‐off for AST and ALT of 40 IU/L, to allow comparison with the literature. The lower limits of normal for albumin were taken as 35 g/L, platelets 150 × 10^9^/L.^12^

Liver biopsy scoring systems Knodell and Metavir were used for liver fibrosis staging, which was categorized as no fibrosis, portal fibrosis (Knodell fibrosis score 1, Metavir F1), bridging or significant fibrosis (Knodell fibrosis score 3, Metavir F2‐3) and cirrhosis (Knodell fibrosis score 4, Metavir F4).^13^, ^14^ Liver biopsies were reviewed at each centre by experienced histopathologists.

Results of liver stiffness evaluated by transient elastography (TE) were categorized as normal (<5.0 kPa), mild increase (5.0 to <7.0 kPa), moderate (7.0 to <9.0 kPa) and severe increase (≥9.0 kPa); ≥9 kPa was taken as predictor of severe fibrosis (≥F3).^15^, ^16^

Successful outcome was defined as sustained virological response 24 weeks after the end of treatment (SVR24).

Heights were converted to height‐for‐age *z*‐scores (HAZ) using the WHO Growth Standard for measurements at age <5 years old,^17^ and WHO 2007 growth reference at age 5‐18 years.^18^

### 2.2 Statistical analysis

Categorical data were summarized as frequency (percentage) and continuous data as median [interquartile range (IQR)] or means (standard deviations, SD). Univariable comparisons of characteristics according to last liver biopsy result, last TE result and SVR24 were assessed using the chi‐square, Fisher's exact, Mann‐Whitney or Kruskal‐Wallis test, as appropriate. In analyses of age and duration of infection, the continuous forms of the variables were used to determine whether age differed across groups. Correlation between levels of liver stiffness from TE and degree of fibrosis measured by liver biopsy was assessed in children who had both procedures within a 12‐month period using Kendall's Tau‐b correlation coefficient. Multivariable analysis of liver stiffness from all TE scans was carried out using a linear random effects model to allow for correlation in repeated measurements in individuals. The model assessed the association between age at TE, sex, genotype, centre and mode of infection.

Analyses were conducted using STATA/IC 15.1 (Stata Corp, College Station, TX, USA).

## 3 RESULTS

A total of 301 children under follow‐up were included 101 were from Moscow, 100 from St Petersburg and 100 from Krasnoyarsk (Supporting information Table S1). Half were male (156, 52%), most were of white ethnicity (281, 93%), and born in Russia (297, 99%). The most common mode of infection was vertical (196, 65%) followed by suspected healthcare‐associated infection (70, 23%; of these 39 were reported to be linked to receipt of blood products, 31 had a history of invasive procedures with three patients having a history of cancer); the rest were one injecting drug use and 34 (11%) with unknown modes of acquisition. Median age at diagnosis of HCV infection was 1.7 years [IQR, 0.7, 3.7] in those vertically infected, 9.2 [6.2, 12.6] years in those with suspected healthcare‐associated infection and 8.2 [4.4, 13.1] years in those with other or unknown mode of infection. Most children had genotype 1b (155, 51%) or 3 (111, 37%). A higher proportion of nonvertically infected children were clinically symptomatic at HCV testing, had elevated liver transaminases or were screened before invasive procedures (Supporting information Table S2).

At last follow‐up, in children with vertical, suspected healthcare‐associated and other/unknown modes of infection, respectively, median age was 8.9 [6.0, 11.8], 15.2 [12.7, 17.3] and 12.5 [10.7, 17.5] years and duration of diagnosed infection was 6.0 [3.0, 8.4], 4.2 [2.5, 7.9] and 3.5 [1.8, 7.0]. Six vertically infected children were HIV‐coinfected; one with suspected nosocomial infection was HBV‐coinfected.

### 3.1 Laboratory investigations

At last visit, 82 (27%) children had ALT >40 IU/L, 88 (29%) AST >40 IU/L (108 (36%) had ALT >40 IU/L or AST >40 IU/L), six (2%) had platelets <150 × 10^9^/L and 15 (5%) had albumin <35 g/L. No differences were observed by duration of infection/age in vertically infected children or duration of diagnosed infection in nonvertically infected.

### 3.2 Liver ultrasound

All children had liver ultrasound results reported. At last, ultrasound 171 (57%) had normal liver, 113 (38%) had increased echogenicity, 96 (32%) heterogeneous structure (86 (29%) had both), 11 (4%) had steatosis and 15 (5%) had signs of portal hypertension.

### 3.3 Liver biopsy

Ninety‐two (31%) children had a liver biopsy (four children had two), with median age at last biopsy of 9.4 [IQR 6.1, 13.0] years (Table 1). The likelihood of having had a liver biopsy increased with age; at last follow‐up six of 52 (12%) children aged <5 years had had a biopsy compared to 27 of 102 (26%) 6‐ to 10‐year‐olds, 31 of 92 (34%) 11‐ to 15‐year‐olds and 28 of 55 (51%) aged >15 years (*P* < 0.001). Forty‐two (21%) vertically infected children had a biopsy vs 50 (48%) of those with nonvertical infection but this difference was nonsignificant after adjusting for age (*P* = 0.898). There was significant variation by centre with 68 (67%) of children at the Moscow centre having had a biopsy compared to 11 (11%) in St Petersburg and 13 (13%) in Krasnoyarsk (*P* < 0.001).

**Table 1:** Bridging fibrosis and cirrhosis detected on liver biopsy by patient characteristics

	All with liver biopsy	No bridging fibrosis	Bridging fibrosis or cirrhosis	*P*‐value
	N (%) or median [IQR]			
All	92	53 (58)	39 (42)	
Sex				
Male	44 (48)	24 (55)	20 (45)	0.569
Female	48 (52)	29 (60)	19 (40)	
Mode of infection				
Vertically infected	42 (46)	27 (64)	15 (36)	0.235
Not vertically infected	50 (54)	26 (52)	24 (48)	
HCV genotype				
1a/b	64 (70)	35 (55)	29 (45)	0.391
2/3	28 (30)	18 (64)	10 (36)	
Centre				
Moscow	68 (74)	38 (56)	30 (44)	0.181
St Petersburg	11 (12)	9 (82)	2 (18)	
Krasnoyarsk	13 (14)	6 (46)	7 (54)	
All patients				
Age at biopsy, Median [IQR]	9.4 [6.1, 13.0]	8.8 [6.0, 12.2]	10.2 [7.1, 14.1]	0.179
≤5 y	21 (23)	13 (62)	8 (38)	
6‐10 y	35 (38)	21 (60)	14 (40)	
11‐15 y	27 (29)	14 (52)	13 (48)	
>15 y	9 (10)	5 (56)	4 (44)	
Vertically infected (n = 42)				
Duration of infection/age at biopsy, Median [IQR]	6.3 [4.7, 9.0]	6.1 [4.5, 7.3]	7.8 [5.0, 10.2]	0.185
≤5 y	18 (43)	12 (67)	6 (33)	
6‐10 y	18 (43)	12 (67)	6 (33)	
>10 y	6 (14)	3 (50)	3 (50)	
Nonvertically infected (n = 50)				
Age at biopsy, Median [IQR]	11.8 [9.3, 14.8]	11.8 [9.2, 14.8]	11.7 [9.5, 14.8]	0.892
≤5 y	3 (6)	1 (33)	2 (67)	
6‐10 y	17 (34)	9 (53)	8 (47)	
11‐15 y	22 (44)	12 (55)	10 (45)	
>15 y	8 (16)	4 (50)	4 (50)	
Duration of diagnosed infection at biopsy, Median [IQR]	1.9 [0.5, 8.8]	6.2 [1.2, 10.5]	0.6 [0.4, 2.9]	0.019
≤5 y	31 (62)	12 (39)	19 (61)	
6‐10 y	11 (22)	9 (82)	2 (18)	
>10 y	8 (16)	5 (63)	3 (27)	

IQR, interquartile range.

At last biopsy, 38 (41%) had signs of bridging fibrosis; median age at diagnosis was 10.4 [7.1, 14.1] years, with range of 2.1‐17.8 years. Cirrhosis was identified at liver biopsy in one 15‐year‐old adolescent in whom infection acquired through contaminated blood products was diagnosed 7 months prior to biopsy. Rates of bridging fibrosis or cirrhosis at the last biopsy are summarized in Table 1.

### 3.4 Transient elastography

**Figure 1:**
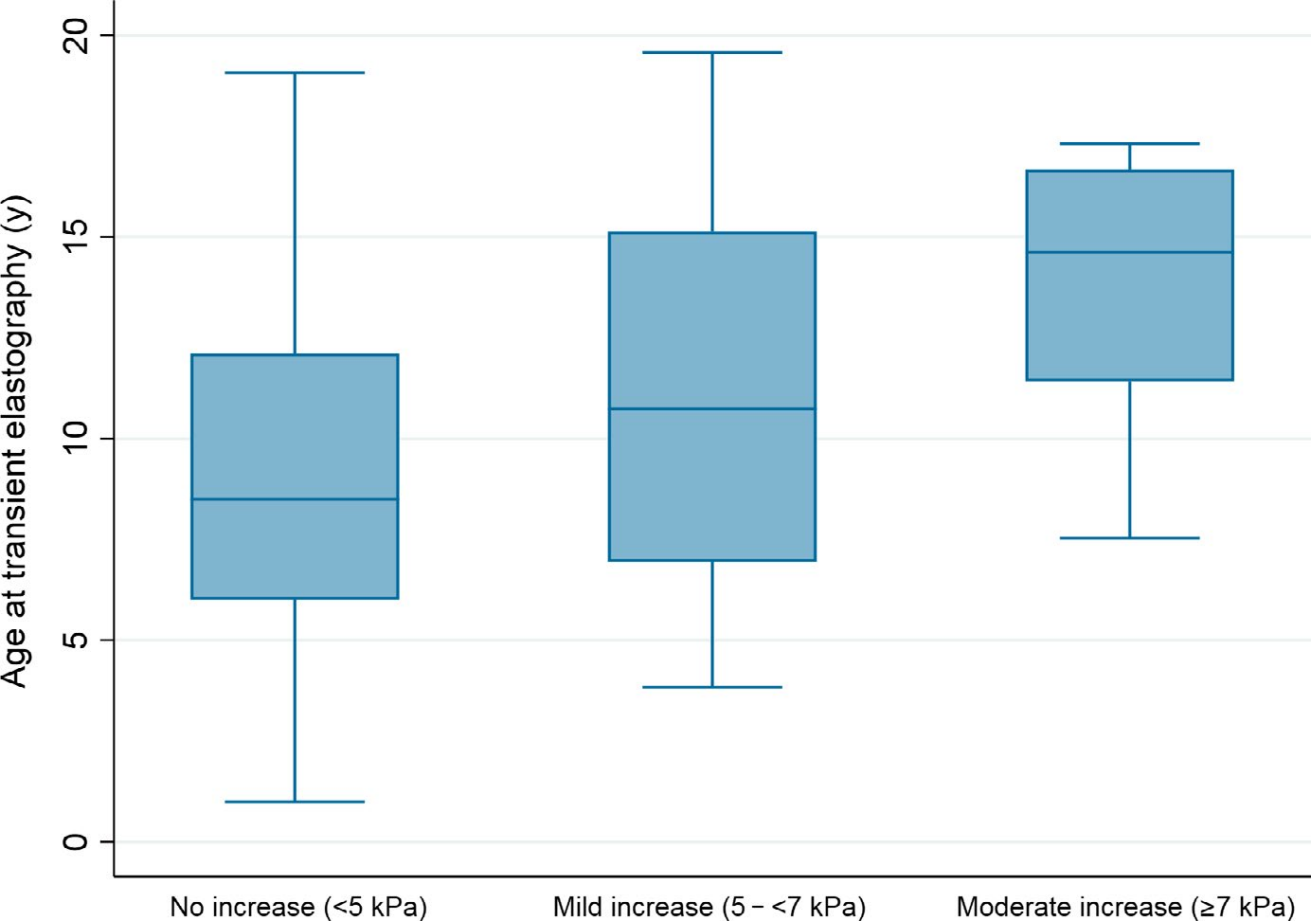
Age at transient elastography (TE) by degree of liver stiffness. Association between age and liver stiffness measured using TE in 223 children with HCV in Russia. There was a significant positive association between age and liver stiffness (*r* = 0.22, *P* = 0.001)

A total of 345 TE measurements were reported in 223 (74%) children. Results of the last TE are summarized in Table 2. There was a significant association between older age at TE and increased liver stiffness (*r* = 0.22, *P* = 0.001) (Figure 1). Moderate increase in liver stiffness (≥7 kPa) was not seen in children <7.5 years of age.

**Table 2:** Increased liver stiffness detected on TE by patient characteristics

	All with TE	No increase (<5 kPa)	Mild increase (5 to <7 kPa)	Moderate or severe increase (≥7 kPa)	*P*‐value
	n (%) or median [IQR]				
All patients	223	156 (70)	59 (26)	8 (4)	
Sex					
Male	110 (49)	84 (76)	22 (20)	4 (4)	0.096
Female	113 (51)	72 (64)	37 (33)	4 (4)	
Mode of infection					
Vertically infected	160 (72)	119 (74)	36 (23)	5 (3)	0.072
Not vertically infected	63 (28)	37 (59)	23 (37)	3 (5)	
HCV genotype					
1a/b	121 (55)	86 (71)	31 (26)	4 (3)	0.929
2/3	100 (45)	69 (69)	27 (27)	4 (4)	
Centre					
Moscow	61 (27)	30 (49)	24 (39)	7 (11)	<0.001
St Petersburg	70 (31)	52 (74)	17 (24)	1 (1)	
Krasnoyarsk	92 (41)	74 (80)	18 (20)	0	
All patients					
Age at the last TE, Median [IQR]	9.0 [6.3, 13.2]	8.5 [6.0.12.1]	10.7 [6.9, 15.1]	14.6 [11.4, 16.7]	0.003
≤5 y	51 (23)	39 (76)	12 (24)	0	
6‐10 y	88 (39)	68 (77)	18 (20)	2 (2)	
11‐15 y	53 (24)	33 (62)	17 (32)	3 (6)	
>15 y	31 (14)	16 (52)	12 (38)	3 (10)	
Vertically infected (n = 160)					
Duration of infection/age at the last TE, Median [IQR]	7.7 [5.5, 11.0]	7.6 [5.4, 9.9]	8.0 [5.3, 12.9]	13.2 [9.6, 14.1]	0.066
≤5 y	48 (30)	37 (77)	11 (23)	0	
6‐10 y	72 (45)	57 (79)	13 (18)	2 (3)	
11‐15 y	31 (19)	22 (71)	7 (23)	2 (6)	
>15 y	9 (6)	3 (33)	5 (56)	1 (11)	
Nonvertically infected (n = 63)					
Age at the last TE, Median [IQR]	13.2 [9.4, 17.0]	12.8 [8.8, 16.6]	13.5 [9.5, 17.0]	16.9 [15.1, 17.3]	0.353
≤5 y	3 (5)	2 (67)	1 (33)	0	
6‐10 y	16 (25)	11 (69)	5 (31)	0	
11‐15 y	22 (35)	11 (50)	10 (45)	1 (5)	
>15 y	22 (35)	13 (59)	7 (32)	2 (9)	
Duration of diagnosed infection at the last TE, Median [IQR]	8.5 [2.8, 12.8]	8.5 [3.4, 12.1]	8.6 [1.8, 13.1]	2.7 [0.6, 9.1]	0.429
≤5 y	25 (40)	15 (60)	8 (32)	2 (8)	
6‐10 y	19 (30)	10 (53)	8 (42)	1 (5)	
>10 y	12 (19)	7 (58)	5 (42)	0	
>15 y	7 (11)	5 (71)	2 (29)	0	
Treatment status at the last TE (n = 223)					
Not commenced	126 (57)	89 (71)	35 (28)	2 (2)	0.026
Treatment ongoing	15 (7)	6 (0)	8 (53)	1 (7)	
Treatment complete	82 (37)	61 (74)	16 (20)	5 (6)	

IQR, interquartile range; TE, transient elastography.

In 84 children with >1 TE, median time between first and last TE was 18.7 [6.4, 19.5] months. In patients with ≥1 TE who completed treatment before the last TE, mean liver stiffness decreased from 5.2 (SD 1.7) to 4.6 (SD 1.6) between the first and last TE (*P* < 0.001) (Supporting Information Figure S3).

In multivariable random effects analysis of all available TE results, there was no association between mode of infection (*P* = 0.577), sex (*P* = 0.625) and genotype (*P* = 0.269) but for every 1 year increase in age at TE mean stiffness increased by 0.09 kPa (95% CI 0.05, 0.13, *P* < 0.001). Mean liver stiffness was 1.1 kPa (0.7, 1.5, *P* < 0.001) lower at both St Petersburg and Krasnoyarsk centres than in Moscow.

### 3.5 Comparison of biopsy and transient elastography results

**Figure 2:**
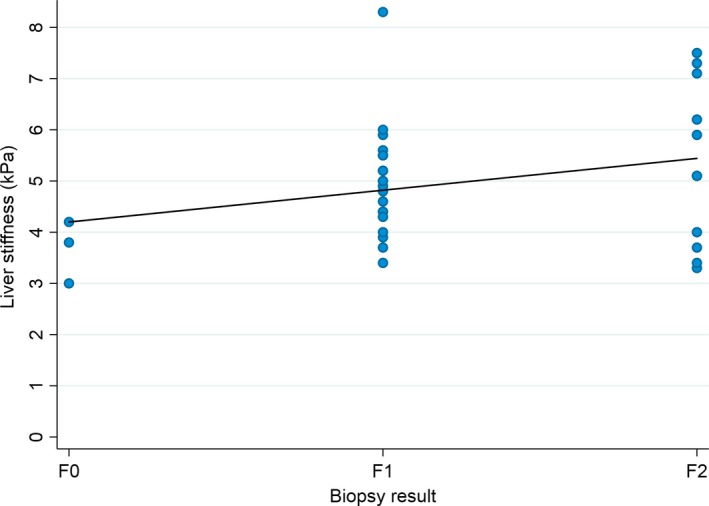
Comparison of liver stiffness measured using transient elastography (TE) and fibrosis stage from liver biopsy. Association between liver stiffness measured using TE and fibrosis stage determined from liver biopsy in 34 children with HCV in Russia who had a liver biopsy within 1 y of a TE. There was a significant correlation between liver stiffness and biopsy results (Tau‐b = 0.29, *P* = 0.042)

Sixty‐four children had both a biopsy and a scan. For 34, scanning occurred within a year of a biopsy (Figure 2), with a significant but relatively weak correlation between TE and biopsy results (Tau‐b = 0.29, *P* = 0.042). In the three patients with fibrosis stage F0, TE ranged from 3 to 4.2 kPa. In 20 patients with stage F1 median stiffness was 4.9 [4.4, 5.5], range 3.4‐8.3 kPa and in 11 patients with stage F2 was 5.4 [4.0, 7.1], range 3.3‐7.5 kPa. No children with F3 or F4 had TE results reported.

### 3.6 Treatment

Overall, 205 (68%) children received treatment, most commonly pegylated interferon (PegIFN) alfa‐2b and ribavirin (RBV); 37 (18%) received >1 treatment course. Median duration was 16 [14, 28] months. Indications for the last treatment were evidence of fibrosis on liver biopsy or TE in 62 (30%), elevated ALT with no fibrosis in 60 (29%) and family preference in 83 (40%). Most treated children (191/205, 93%) experienced adverse reactions, most commonly flu‐like symptoms (Table 3). Six children required treatment with antidepressants and one required thyroxin replacement. Across all 205 treated children, 54 (26%) had dose reduction and/or discontinuation. Thirty‐nine (19%) children prematurely discontinued the last course of treatment: 33 (16%) for inadequate response and 6 (3%) for drug reactions.

**Table 3:** Treatment duration, discontinuations, adverse events and outcomes

	All	PegIFN alfa‐2b and ribavirin	IFN alfa‐2a/b plus ribavirin	IFN alfa‐2a/b only	Other
	n (%) or median [IQR]				
Total	205	114	13	59	19
Duration of treatment (mo)					
All	16 [14, 28]	21 [14, 28]	17 [14, 18]	16 [14, 28]	15 [11, 27]
Genotype 1a/b	28 [16, 28]	28 [28, 28]	16 [14, 23]	17 [15, 28]	27 [8, 28]
Genotype 2/3	14 [14, 15]	14 [14, 14]	15 [14, 17]	15 [14, 26]	15 [14, 16]
Treatment prematurely discontinued	39 (19)	12 (11)	3 (23)	18 (31)	6 (32)
Reasons for premature discontinuation^a^					
Inadequate response	33 (85)	8 (67)	2 (67)	17 (94)	6 (100)
Drug reactions	6 (15)	5 (42)	0	0	1 (17)
Family decision	8 (21)	4 (33)	2 (67)	2 (11)	0
Side effects^a^					
None	14 (7)	1 (1)	2 (15)	10 (17)	1 (5)
Hypersensitivity reaction	6 (3)	6 (5)	0	0	0
Diarrhoea	6 (3)	5 (4)	0	1 (2)	0
Nausea/vomiting	20 (10)	17 (15)	1 (8)	2 (3)	0
Hepatotoxicity	4 (2)	2 (2)	0	1 (2)	1 (5)
Hypothyroidism	2 (1)	2 (2)	0	0	0
Hair loss	39 (19)	34 (30)	1 (8)	4 (7)	0
Poor weight gain/weight loss	48 (23)	36 (40)	0	10 (17)	2 (11)
Insomnia	34 (17)	26 (22)	1 (8)	7 (12)	0
Depression/anxiety	32 (16)	19 (17)	1 (8)	10 (17)	2 (11)
Flu‐like symptoms	186 (91)	110 (96)	11 (85)	48 (81)	17 (89)
Weakness	79 (39)	54 (47)	3 (23)	19 (32)	3 (16)
Local reaction at injection site	114 (56)	74 (65)	1 (8)	25 (42)	14 (74)
Anaemia	46 (22)	34 (30)	0	10 (17)	2 (11)
Thrombocytopenia	21 (10)	14 (12)	0	4 (7)	0
Neutropenia	59 (29)	40 (35)	0	15 (25)	4 (21)
Other	21 (10)	19 (17)	0	2 (3)	0
SVR24					
All	100 (49)	65 (57)	6 (46)	22 (37)	7 (37)

IQR, interquartile range.

More than one adverse event or discontinuation reason may be reported in the same child. Multiple adverse events were reported in the six children who discontinued due to drug reactions including hypersensitivity (n = 2), weakness (n = 2), depression/anxiety (n = 4), anaemia (n = 2), alopecia (n = 3) and insomnia (n = 1).

HCV results 24 weeks post‐treatment discontinuation were available for all treated patients, 100 (49%) of whom had SVR24 (Table 3). SVR24 was achieved in 6 children (46%) treated with IFN alfa‐2a/b/RBV, 22 (37%) treated with IFN alfa‐2a/b only and 7 (37%) on other treatments (Table 3). Of those treated with PegIFN alfa‐2b/RBV, 65 (57%) achieved SVR24 overall (35 (55%) with GT1, and 30 (61%) with GT2/3) (Table 4). SVR24 was more common in those vertically infected (*P* = 0.014), but the difference disappeared after adjusting for age at treatment start (*P* = 0.216). Nonvertically infected children also had a higher rate of treatment discontinuation (Supporting information Table S2). Treatment outcomes varied significantly by centre, with the lowest proportion of patients with SVR24 in Moscow (34%) and the highest in Krasnoyarsk (74%) (Supporting information Table S1). The Moscow cohort had more complex patients, more patients diagnosed at older ages and more with clinical symptoms or elevated transaminases at diagnosis. More patients in Moscow also had increased liver stiffness measured by TE and bridging fibrosis on liver biopsy suggesting more progressive disease (Tables 1 and 2; Supporting information Table S1). Higher treatment discontinuations in the Moscow cohort (31% vs 15% St Petersburg and 8% Krasnoyarsk) (Supporting information Table S1) also partially explain the centre differences in cure rates.

**Table 4:** SVR24 by patient characteristics in all treated children and those treated with PegIFN alfa‐2b + ribavirin only

	ALL treated				PegIFN alfa‐2b + ribavirin only		
	n (%)	No SVR24	SVR24	*P*‐value	No SVR24	SVR24	*P*‐value
All	205	105 (51)	100 (49)		49 (43)	65 (57)	
Sex							
Male	102 (50)	58 (57)	44 (43)	0.108	25 (43)	33 (57)	0.979
Female	103 (50)	47 (46)	56 (54)		24 (43)	32 (57)	
Mode of infection							
Vertically infected	126 (61)	56 (44)	70 (56)	0.014	36 (44)	45 (56)	0.621
Not vertically infected	79 (39)	49 (62)	30 (38)		13 (39)	20 (61)	
HCV genotype^a^							
1a/b	125 (61)	69 (55)	56 (45)	0.129	29 (45)	35 (55)	0.486
2/3	79 (39)	35 (44)	44 (56)		19 (39)	30 (61)	
Multiple treatments							
No	186 (82)	83 (49)	85 (51)	0.268	37 (42)	52 (58)	0.566
Yes	37 (18)	22 (59)	15 (41)		12 (48)	13 (52)	
All patients							
Age at treatment start, median [IQR]	8.9 [5.8, 12.9]	10.0 [6.7, 14.1]	8.2 [4.7, 12.0]	0.014	10.0 [7.0, 14.1]	8.6 [6.1, 12.7]	0.435
<2 y	10 (5)	2 (20)	8 (80)		0	0	
2‐5 y	45 (22)	20 (44)	25 (56)		9 (36)	16 (64)	
6‐10 y	73 (36)	36 (49)	37 (51)		18 (42)	25 (58)	
>10 y	77 (38)	47 (61)	30 (39)		22 (48)	24 (52)	
Vertically infected (n = 126)							
Duration of infection/age at treatment start, median [IQR]	7.1 [4.4, 10.0]	7.5 [4.9, 11.3]	6.5 [4.2, 9.1]	0.146	8.7 [6.5, 11.6]	7.1 [4.7, 9.8]	0.102
<2 y	10 (8)	2 (20)	8 (80)		0	0	
2‐5 y	40 (32)	17 (43)	23 (58)		7 (30)	16 (70)	
6‐10 y	48 (38)	22 (46)	26 (54)		16 (46)	19 (54)	
>10 y	28 (22)	15 (54)	13 (46)		13 (57)	10 (43)	
Nonvertically infected (n = 79)							
Age at treatment start, median [IQR]	11.9 [8.8, 16.4]	12.9 [9.4, 15.9]	11.7 [8.7, 16.4]	0.824	14.1 [10.8, 15.5]	14.1 [10.8, 16.5]	0.854
2‐5 y	5 (6)	3 (60)	2 (40)		2 (100)	0	
6‐10 y	25 (32)	14 (56)	11 (44)		2 (25)	6 (75)	
>10 y	49 (62)	32 (65)	17 (35)		9 (39)	14 (61)	
Duration of diagnosed infection at treatment start, median [IQR]	1.6 [0.7, 6.0]	1.4 [0.6, 3.4]	2.3 [1.1, 6.6]	0.047	1.4 [1.0, 4.7]	2.3 [1.2, 8.3]	0.407
<2 y	43 (54)	31 (72)	12 (28)		9 (53)	8 (47)	
2‐5 y	16 (20)	8 (50)	8 (50)		1 (25)	3 (75)	
6‐10 y	16 (20)	7 (44)	9 (56)		1 (11)	8 (89)	
>10 y	4 (5)	3 (75)	1 (25)		2 (67)	1 (33)	

IQR, interquartile range.

a Genotype unknown for one patient.

Of the 205 treated children, 193 had height data at the start and end of treatment. At treatment start, mean HAZ was −0.03 (SD 1.13) and *z*‐scores decreased during treatment (mean change −0.11 [95% CI −1.18, −0.29]). In children on PegIFN alfa‐2b/RBV mean *z*‐score was −0.12 (1.15) and decreased by −0.09 (95% CI −0.19, 0.01) during treatment. Change in *z*‐scores did not vary significantly by treatment type (*P* = 0.326). The largest decreases in *z*‐scores were observed in children aged 10‐15 years (−0.17 [95% CI −0.23, −0.09]) followed by 6‐ to 10‐year‐olds (−0.12 [−0.23, −0.02]) and under 5s (−0.10 [−0.42, 0.22]) with no change in those 15 years or more at treatment start (0.05 [−0.02, 0.12]).

## 4 DISCUSSION

Russia has one of the largest populations of children living with HCV in Europe; however there are scarce data on CHC in children in peer‐reviewed literature published outside Russia. This study contributes to addressing this knowledge gap by providing detailed characterization of a large number of children and adolescents with CHC, of whom 65% were vertically infected.

The most frequent reason for HCV testing was HCV infection in a family member, usually the mother, which coincided with the predominance of vertical mode of transmission in the study and with national surveillance data.^6^ Vertical transmission of HCV so far remains the only nonpreventable route of acquisition of hepatitis C infection in children. Notably, the observed proportion of vertical infection is lower here compared to that reported in western European children.^19^

The finding that infection possibly acquired via transfusion of blood components or through invasive procedures comprised nearly a quarter of all cases here is important. In the national surveillance report, a similar proportion of children had unknown mode of transmission.^6^ A recent outbreak of hospital‐acquired HCV infection at a Russian children's hospital in the Amur region highlights the significance of the problem.^20^ Healthcare‐associated HCV infections are almost impossible to confirm retrospectively. History of invasive healthcare procedures, when vertical transmission is ruled out, can be considered as a proxy of healthcare‐associated infections in children and younger adolescents, as other routes of transmission such as cosmetic procedures (piercing and tattooing), sexual transmission and injecting drug use, are extremely rare in this age group. Suspected healthcare‐associated HCV infections in children can serve as a sentinel of possible ongoing transmission of blood‐borne pathogens in health care settings.

Distribution of HCV genotypes in children here was similar to the distribution reported in the national reports,^6^, ^8^ with GT1b being a predominant genotype, followed by GT3. Genotype distribution in adults is associated with mode of transmission, with GT1a, 3a and 4 being mostly IDU related and GT1b and 2 associated with blood transfusion and unsafe medical procedures.^21^

Among those with liver biopsy, we found liver disease progression, including to bridging fibrosis in 41% of children and cirrhosis in one 15‐year‐old adolescent, and nearly half of children aged older than 10 years had bridging fibrosis. Notably, children as young as 2‐5 years of age had bridging fibrosis highlighting rapid progression at young age in a minority. As determinants of liver disease progression are uncertain, genetic and other host factors could have had an impact on the particularly aggressive course of disease in some children. Only a third of children had a liver biopsy, potentially reflecting selection bias of choosing children with suspected advanced liver disease for the invasive staging. However, in our study TE was introduced from 2008 to 2011 in different centres, and prior to this liver biopsy was the only method of staging liver disease. Differences in local practices and complexity of patients likely played a role as most children with liver biopsy attended the Moscow centre. We did not find a relationship between age and advanced liver disease; however, likelihood of liver biopsy increased with age and it is likely that younger children undergoing biopsies did so based on non‐invasive tests suggestive of liver disease. Overall, high proportions of children with bridging fibrosis on liver biopsy here indicate that a substantial proportion of children with CHC have advanced liver disease.

Three‐quarters of children had evaluation of liver stiffness by TE, used as indirect assessment of liver fibrosis. Greater liver stiffness was observed with older age and duration of infection, although only two (1%) children had liver stiffness indicative of advanced fibrosis (≥9 kPA) and six (3%) had a moderate increase (7 to <9 kPa); 7% of children over 10 years of age had a moderate or severe increase. The paired TE and liver biopsy results were available for children with initial stages of liver disease (F0‐F2); no children with F3 or F4 had TE as all of them had liver biopsy before TE was introduced in the centres. This could be a possible explanation for the weak correlation between TE and liver biopsy results here. Drawbacks of using TE for evaluation of liver disease staging have high inter‐patient variation, lack of correlation between TE and liver biopsy for nonadvanced liver fibrosis and absence of validated data for children with CHC.^22^ High inter‐ and intra‐observer variability of liver biopsy evaluation^23^ and different biopsy scoring systems add to these limitations. At present, it is not possible to confidently identify rapid CHC progressors with non‐invasive tests, and liver biopsy remains a gold standard for liver disease staging, despite being an invasive, expensive and operator‐dependant procedure requiring technical and pathology expertise and general anaesthetic in younger children. There is a pressing need of a large high‐quality study validating TE against liver biopsy in paediatric population with HCV. Our dataset was insufficient to address this. A larger sample of paired TE and liver biopsy across all stages of liver fibrosis in children with HCV is required, and a pooled analysis of data from different cohorts may be necessary. A Russian European alliance for research among women, children and adolescents impacted by HIV, TB and HCV (REACH) aims to address this knowledge gap.^24^

Overall, our study showed that a higher proportion of children than previously thought may develop advanced liver disease with rapid progression in some children. These findings, together with a few recent observational studies,^25^, ^26^ challenge the traditional belief that HCV causes no health problems in childhood. Our study confirms results of other small paediatric studies that older age and duration of infection are associated with higher risk of liver disease progression.^27^, ^28^, ^29^ Recently presented national data from the UK highlighted poor mid‐term prognosis in individuals who acquired HCV in childhood with 33% progressing to cirrhosis by median age of 33 years.^30^ Liver disease progression in childhood or young adulthood in a substantial proportion of patients with HCV acquired in childhood and the inability to identify rapid progressors are strong arguments for treating all children and adolescents provided effective and well‐tolerated treatments are available.

PegIFN‐based treatments remain the only currently available treatment for children in Russia. PegIFN/RBV treatment is prolonged (24 weeks for HCV GT2,3 and 48 weeks for other genotypes) and invasive (requiring weekly injections of PegIFN), and although it is reported to be better tolerated by children compared to adults,^31^ it is associated with substantial toxicity and has suboptimal efficacy. Over two‐thirds of children in this study were treated. Only 57% of children treated with PegIFN/RBV achieved SVR24, similar to the results of a meta‐analysis demonstrating 58% of overall SVR across genotypes; rates of treatment discontinuation due to adverse events were also similar.^31^ The proportion of children with SVR24 was lowest in Moscow, which might be explained by their more complex patients, and also their treatment of children from across Russia, with joint follow‐up with local hepatitis C clinics potentially contributing to more frequent treatment discontinuations. This highlights the difficulties of treating children with prolonged IFN‐based schemes, especially those living in remote settings.

Most treated children in our study experienced adverse events. Of those treated with PegIFN/RBV, one in three developed anaemia or neutropenia, one in three had hair loss and one in six suffered from anxiety/depression; the latter may be underestimated given that young children do not understand or communicate these adverse events well. Consistent with previous studies,^32^, ^33^, ^34^ we showed that growth was adversely affected during treatment, particularly among young adolescents.

Our results confirm that currently used regimens have suboptimal outcomes and very poor safety profiles. Today, when DAAs can achieve cure rates of more than 95%, it seems unethical to treat patients with IFN‐based regimens with suboptimal response rates and nearly universal occurrence of adverse reactions as indicated by a 93% rate in this study. However, only IFN‐based regimes are currently approved for paediatric treatment in Russia. For DAAs to be licensed in children, it is required that paediatric studies are conducted in Russia which presents an additional hurdle to making DAAs accessible to children. Clinicians, regulatory authorities and the community health sector should work together to expedite the process. Outside Russia, at present only two drugs (sofosbuvir and ledipasvir) are licensed for adolescents aged ≥12 years by the FDA and EMA; paediatric access to these drugs is delayed in most countries, with prohibitive costs. Middle‐income countries are in a particularly difficult situation as they are excluded from voluntary licensing agreements and compulsory licensing is challenging to implement.^35^, ^36^ We showed that patients completing treatment had a significant decrease of liver stiffness measured by TE, suggesting that treatment in childhood maybe beneficial. In the absence of DAA‐regimens for children, the only treatment option in many countries are IFN‐based schemes. So, the ethical question remains whether children with evidence of liver disease progression should wait for new regimens or be treated now with suboptimal but available treatment. The options of treating children with DAAs within prospective studies should be implemented where possible.

Antenatal HCV screening is routine in Russia^6^ and presents a unique opportunity for timely intervention to cure maternal HCV and prevent vertical transmission. With currently available DAAs, which have an excellent safety profile, this could become a promising component within a broader strategy for eliminating paediatric HCV. Results from a phase I safety trial of sofosbuvir and ledipasvir for pregnant women with GT1 are expected in 2019,^37^ and a strategy trial is required.

This large study of children and adolescents with chronic HCV in Russia highlights the important burden of disease in this setting and the need to treat children early to prevent progression of liver disease with more effective and better‐tolerated treatment. Although treatment coverage was high, only half of those treated achieved SVR24. There is therefore an urgent need to speed up access to new DAAs, particularly for older children and adolescents, and make it available at affordable prices in all settings.

## AUTHORS’ CONTRIBUTION

Study concept and design: AT, GI, CT; Acquisition of data: GVV, TAS, VNP, NVR, ELT; Analysis and interpretation of data: AT, GVV, SC, AIK, GI, CT; Drafting of the manuscript: AT, SC, GI, CT; Critical revision of the manuscript for important intellectual content: GVV, TAS, VNP, NVR, AIK, ELT; Statistical analysis: SC; Obtained funding: AT, CT; Study supervision: AT, GVV, CT; All authors approved the submitted manuscript and agreed to be accountable for all aspects of all aspects of the work.

## CONFLICTS OF INTEREST

None.
