# Supplementary material for: Advanced liver disease in Russian children and adolescents with chronic hepatitis C
Source: J Viral Hepat. 2019 Apr 7;26(7):881–92. doi: 10.1111/jvh.13093 (PMC7155091; doi:10.1111/jvh.13093)
Supplement: Supplementary file 1 [file JVH-26-881-s001.docx]

**S1 Table: Characteristics of children with HCV infection by centre**

|  | All | Moscow | St Petersburg | Krasnoyarsk | P-value |
| --- | --- | --- | --- | --- | --- |
|  | N (%) or median [IQR] | | | |  |
| All | 301 | 101 | 100 | 100 |  |
| Male sex | 156(52) | 51(51) | 47(47) | 58(58) | 0.282 |
| Mode of infection |  |  |  |  |  |
| Vertically infected | 196(65) | 47(47) | 82(82) | 67(67) | <0.001 |
| Suspected healthcare-associated | 70(23) | 45(45) | 9(9) | 16(16) |  |
| Injecting drug use | 1(0) | 1(1) | 0 | 0 |  |
| Other/Unknown | 34(11) | 8(8) | 9(9) | 17(17) |  |
| HCV genotype |  |  |  |  |  |
| 1a | 10(3) | 4(4) | 6(6) | 0(0) | <0.001 |
| 1b | 155(51) | 58(57) | 38(38) | 59(59) |  |
| 2 | 15(5) | 6(6) | 3(3) | 6(6) |  |
| 3 | 111(37) | 33(33) | 44(44) | 34(34) |  |
| Unknown | 10(3) | 0 | 9 (9) | 1(1) |  |
| Age at diagnosis of HCV | 3.1[1.1,8.2] | 6.2[2.5,9.9] | 1.1[0.4,4.6] | 3.1[1.7,7.0] | <0.001 |
| Age at last follow up | 10.8[7.4,14.7] | 12.1[7.6.15.2] | 10.5[7.6,13.5] | 10.0[7.0,14.6] | 0.185 |
| Reason for HCV testing† |  |  |  |  |  |
| Family member with HCV | 186(62) | 43(43) | 76(76) | 67(67) | <0.001 |
| History of blood transfusion | 33(11) | 25(24) | 2(2) | 6(6) | <0.001 |
| History of invasive procedures | 25(8) | 11(11) | 1(1) | 13(13) | 0.002 |
| Clinical symptoms | 29(10) | 24(24) | 3(3) | 2(2) | <0.001 |
| *Hepatomegaly* | *15(52)* | *10(42)* | *3(100)* | *2(100)* |  |
| *Hepatosplenomegaly* | *4(14)* | *4(17)* | *0* | *0* |  |
| *Other/Unknown* | *10(34)* | *10(42)* | *0* | *0* |  |
| Elevated transaminases | 84(28) | 54(53) | 27(27) | 3(3) | <0.001 |
| Screening prior to invasive procedure or operation | 29(10) | 26(26) | 2(2) | 1(1) | <0.001 |
| Other | 29(10) | 14(14) | 1(1) | 14(14) | <0.001 |
| Parent known to be infected |  |  |  |  |  |
| Neither | 103(34) | 53(53) | 18(18) | 32(32) | <0.001 |
| Mother only | 164(54) | 41(41) | 60(60) | 63(63) |  |
| Father only | 2(1) | 1(1) | 0 | 1(1) |  |
| Both | 32(11) | 6(6) | 22(22) | 4(4) |  |
| Comorbidities† |  |  |  |  |  |
| None | 211(70) | 72(71) | 80(80) | 59(59) | 0.005 |
| Cardio-respiratory | 25(8) | 12(12) | 5(5) | 8(8) | 0.220 |
| Skin disorders | 12(4) | 6(6) | 2(2) | 4(4) | 0.409 |
| Haematological disorders | 2(1) | 2(2) | 0 | 0 | 0.333 |
| Cancer | 3(1) | 1(1) | 0 | 2(2) | 0.551 |
| Hyperthyroidism | 1(0) | 1(1) | 0 | 0 | 1.000 |
| Nephropathy | 5(2) | 4(4) | 0 | 1(1) | 0.132 |
| Diabetes | 4(1) | 1(1) | 0 | 3(3) | 0.229 |
| Other conditions | 94(31) | 50(50) | 15(15) | 29(29) | <0.001 |
| Raised ALT and/or AST at last visit | 108(36) | 38(38) | 43(43) | 27(27) | 0.056 |
| Patients with ≥1 liver ultrasound | 301(100) | 101(100) | 100(100) | 100(100) |  |
| Patients with ≥1 liver biopsy | 92(31) | 68(67) | 11(11) | 13(13) | <0.001 |
| Patients with ≥1 transient elastography | 223(74) | 61(60) | 70(70) | 92(92) | <0.001 |
| Patients receiving HCV treatment | 205(68) | 80(79) | 60(60) | 65(65) | 0.010 |
| All treated patients with SVR 24 | 100(49) | 27(34) | 25(42) | 48(74) | <0.001 |
| Patients discontinued treatment prematurely | 39(19) | 25(31) | 9(15) | 5(8) | 0.001 |
| Patients who completed a full treatment course with SVR 24 | 93(56) | 27(49) | 19(37) | 47(78) | <0.001 |
| IQR = interquartile range  †More than one reason for testing or comorbidity may be reported for each child | | | | |  |

**S2 Table: Characteristics of children with vertical and non-vertical HCV infection**

|  | All | Vertically infected | Non-vertically infected* | P-value |
| --- | --- | --- | --- | --- |
|  | N (%) or median [IQR] | | | |
| All | 301 | 196 | 105 |  |
| Male sex | 156(52) | 96(49) | 60(57) | 0.177 |
| HCV genotype |  |  |  |  |
| 1a | 10(3) | 8(4) | 2(2) | 0.036 |
| 1b | 155(53) | 88(47) | 67(64) |  |
| 2 | 15(5) | 10(5) | 5(5) |  |
| 3 | 111(38) | 81(43) | 30(29) |  |
| *Unknown* | *10* | *9* | *1* |  |
| Age at diagnosis of HCV | 3.1[1.1,8.2] | 1.7[0.7,3.7] | 9.0[6.1,12.8] | <0.001 |
| Age at last follow up | 10.8[7.4,14.7] | 8.9[6.0,11.8] | 14.7[11.8,17.3] | <0.001 |
| Reason for HCV testing† |  |  |  |  |
| Family member with HCV | 186(62) | 184(94) | 2(2) | <0.001 |
| History of blood transfusion | 33(11) | 0 | 33(31) | <0.001 |
| History of invasive procedures | 25(8) | 0 | 25(24) | <0.001 |
| Clinical symptoms | 29(10) | 10(5) | 19(18) | <0.001 |
| *Hepatomegaly* | *15(52)* | *6(67)* | *9(45)* |  |
| *Hepatosplenomegaly* | *4(14)* | *0* | *4(20)* |  |
| *Other/Unknown* | *9(31)* | *3(33)* | *7(35)* |  |
| Elevated transaminases | 84(28) | 35(18) | 49(47) | <0.001 |
| Screening prior to invasive procedure or operation | 29(10) | 7(4) | 22(21) | <0.001 |
| Other | 29(10) | 6(3) | 23(22) | <0.001 |
| Parent known to be infected |  |  |  |  |
| Neither | 103(34) | 0 | 103(98) | <0.001 |
| Mother only | 164(54) | 164(84) | 0 |  |
| Father only | 2(1) | 0 | 2(2) |  |
| Both | 32(11) | 32(16) | 0 |  |
| Comorbidities† |  |  |  |  |
| None | 211(70) | 151(77) | 60(57) | <0.001 |
| Cardio-respiratory | 25(8) | 11(6) | 14(13) | 0.021 |
| Skin disorders | 12(4) | 7(4) | 5(5) | 0.758 |
| Haematological disorders | 2(1) | 0 | 2(2) | 0.121 |
| Cancer | 3(1) | 0 | 3(3) | 0.042 |
| Hyperthyroidism | 1(0) | 0 | 1(1) | 0.349 |
| Nephropathy | 5(2) | 2(1) | 3(3) | 0.347 |
| Diabetes | 4(1) | 1(1) | 3(3) | 0.124 |
| Other conditions | 94(31) | 44(22) | 50(48) | <0.001 |
| Raised ALT and/or AST at last visit | 108(36) | 66(34) | 42(40) | 0.275 |
| Patients with ≥1 liver ultrasound | 301(100) | 196(100) | 105(100) | NA |
| Patients with ≥1 liver biopsy | 92(31) | 42(21) | 50(48) | <0.001 |
| Patients with ≥1 transient elastography | 223(74) | 160(82) | 63(60) | <0.001 |
| Patients receiving HCV treatment | 205(68) | 126(64) | 79(75) | 0.052 |
| All treated patients with SVR 24 | 100(49) | 70(56) | 30(38) | 0.014 |
| Patients discontinued treatment prematurely | 39(19) | 14(11) | 25(32) | <0.001 |
| Patients who completed a full treatment course with SVR 24 | 93(56) | 65(58) | 28(52) | 0.506 |
| IQR = interquartile range  * Include unknown mode of infection  †More than one reason for testing or comorbidity may be reported for each child | | | | |

**S3: Scatter plot of liver stiffness at the first and last transient elastography by treatment status**

TE = transient elastography, sd = standard deviation
